# Sex‐specific effects of *COMT* Val158Met polymorphism on corpus callosum structure: A whole‐brain diffusion‐weighted imaging study

**DOI:** 10.1002/brb3.786

**Published:** 2017-08-09

**Authors:** Wissam El‐Hage, Helen Cléry, Frederic Andersson, Isabelle Filipiak, Michel Thiebaut de Schotten, Benedicte Gohier, Simon Surguladze

**Affiliations:** ^1^ Université François‐Rabelais de Tours Inserm UMR U930 ‘Imagerie et Cerveau’ Tours France; ^2^ Clinique Psychiatrique Universitaire CHRU de Tours Tours France; ^3^ Inserm 1415 Centre d'Investigation Clinique CHRU de Tours Tours France; ^4^ Inserm U1127 UPMC‐Paris6 UMR‐S 975 CNRS UMR 7225 Brain and Spine Institute Groupe Hospitalier Pitié‐Salpetrière Paris France; ^5^ Brain Connectivity and Behaviour Group Frontlab, Brain and Spine Institute Paris France; ^6^ Department of Psychiatry CHU d'Angers Angers France; ^7^ Institute of Psychiatry, Psychology & Neuroscience King's College London London UK; ^8^ Social & Affective Neuroscience Laboratory Ilia State University Tbilisi Georgia

**Keywords:** *COMT* polymorphism, Diffusion‐weighted imaging (DWI), sex effect, tract‐based spatial statistics (TBSS), white matter

## Abstract

**Background:**

Genetic polymorphisms play a significant role in determining brain morphology, including white matter structure and may thus influence the development of brain functions. The main objective of this study was to examine the effect of Val158Met (rs4680) polymorphism of Catechol‐*O*‐Methyltransferase (*COMT*) gene on white matter connectivity in healthy adults.

**Methods:**

We used a whole‐brain diffusion‐weighted imaging method with Tract‐Based Spatial Statistics (TBSS) analysis to examine white matter structural integrity in intrinsic brain networks on a sample of healthy subjects (*N* = 82).

**Results:**

Results revealed a sex‐specific effect of *COMT* on corpus callosum (CC): in males only, *Val* homozygotes had significantly higher fractional anisotropy (FA) compared to *Met*‐carriers. Volume‐of‐interest analysis showed a genotype by sex interaction on FA in genu and rostral midbody of CC, whereby Val males demonstrated higher FA than Met females.

**Conclusions:**

These results demonstrate the key effect of genes by sex interaction, rather than their individual contribution, on the corpus callosum anatomy.

## 1 INTRODUCTION

Genetic polymorphisms play significant role in determining brain morphology, including white matter structure and may thus influence the development of brain functions. Developmental studies showed that dopamine (DA) is one of the earliest neuromodulators expressed in the developing brain that plays a role in the neuronal maturation and myelination (Bartzokis, 2011; Money & Stanwood, 2013). Increased dopamine presence in the frontal cortex may also cause white matter abnormalities. For instance, mice exposed to a copper chelator cuprizone show a higher level of dopamine in the frontal cortex associated with brain demyelination, myelin breakdown, and loss of oligodendrocytes (Xu et al., 2009; Xu, Yang, McConomy, Browning, & Li, 2010). However, the link between the genetic mechanisms underlying DA functionality and its relationship with white matter integrity in the living human brain remains poorly understood.

One of the most frequently studied dopaminergic genes is Catechol‐*O*‐methyltransferase *(COMT)* gene located within the q11 of the 22nd chromosome (Bertocci et al., 1991; Deutch & Roth, 1990; Grossman, Emanuel, & Budarf, 1992). The *COMT* gene is involved in the metabolic degradation of catecholamines, notably synaptic dopamine, especially in the prefrontal cortex (Männistö & Kaakkola, 1999; Mattay et al., 2003; Tunbridge, Bannerman, Sharp, & Harrison, 2004). This gene is associated with several allelic variants, including the *COMT Val*158*Met* (rs4680), a functional single‐nucleotide polymorphism that codes for a substitution of methionine (*Met*) for valine (*Val*) at codon 158. Previous studies have shown that this *Met* substitution reduces the activity of *COMT* enzyme to one‐quarter of what is originally encoded by the *Val* allele (Lachman et al., 1996; Lotta et al., 1995).

The COMT *Val*158*Met* polymorphism may impact prefrontally mediated cognition (Meyer‐Lindenberg & Weinberger, 2006). Several studies showed that *Met*‐carriers performed better in cognitive tasks that involve prefrontal cortex functionality notably working memory and cognitive flexibility tasks, compared with *Val* homozygotes. This effect was found independently of diagnosis, for example, in healthy controls, individuals with schizophrenia and schizotypal disorder (Goldberg et al., 2003; Malhotra et al., 2002; Minzenberg et al., 2006). However, a meta‐analysis (Barnett, Scoriels, & Munafò, 2008) highlighted that the relationship between *COMT* and cognitive performance was not always evident. More recently, a large population‐based study (Wardle, de Wit, Penton‐Voak, Lewis, & Munafò, 2013) has not found any effect of *COMT* on working memory. The authors suggested that the *COMT* effects are better detectable in neurophysiological experiments rather than in purely cognitive tasks. fMRI studies have indeed demonstrated *COMT* effect on Blood Oxygenation Level Dependent (BOLD) signal in people performing working memory tasks (Egan et al., 2001; Mattay et al., 2003). However, the meta‐analytical data on this topic has been inconsistent. Meta‐analysis by Mier, Kirsch, and Meyer‐Lindenberg (2010) reported significant association between the *COMT* genotype and prefrontal activation in executive function tasks, whereas the most recent meta‐analytical paper indicated that there is presently no (significant) spatial convergence of imaging genetics findings on this association (Nickl‐Jockschat, Janouschek, Eickhoff, & Eickhoff, 2015).

As well as studying the effect of *COMT* on brain functions, the investigators have been increasingly looking at possible associations between *COMT* polymorphisms and brain structure, for example, brain white matter as indexed by fractional anisotropy (FA). Shimony et al. (1999) used quantitative diffusion anisotropy MR images obtained from 13 healthy adults to evaluate white matter integrity. Their results showed that the FA measure reflects density and myelination of fibers, as well as directional coherence, which could contribute to efficient signal transmission. Thomason et al. (2010) examined *COMT* gene‐related differences in four white matter fiber tracts (genu of the corpus callosum, anterior thalamic radiation, inferior fronto‐occipital fasciculus, uncinated fasciculus) in a group of 40 children (aged 9–15). The authors reported a significantly higher FA in *Val*‐allele homozygotes (vs. *Met*/*Val* heterozygotes and *Met* allele homozygotes) in the genu of corpus callosum and anterior thalamic radiation, respectively. Based on animal studies showing increase in DA availability due to inhibition of *COMT* functionality (e.g., Tunbridge et al., 2004), the authors suggested that lower FA measures in *Met*‐carriers were associated with higher dopamine availability that may have inhibited myelination. Their results were in accordance with those of Liu et al. (2010) who employed a haplotype‐based approach to assess the effect of *COMT* gene polymorphisms on white matter integrity. The authors found that the groups with lower *COMT* enzymatic activity (i.e., potentially higher dopamine levels) had lower FA values in the prefrontal white matter tracts.

However, the main effect of *COMT* was not replicated in a study of Kohannim et al. (2012). This voxelwise analysis study reported a combined effect of five genetic SNPs, that is, *COMT Val*158*Met*, clusterin CLU, neuregulin 1 receptor (ErbB4), neurotrophic tyrosine kinase receptor‐type 1 (NTRK1), and the hemochromatosis gene (HFE) which explained ∼6% of the variance in the average FA of the corpus callosum including the genu, body, and splenium in young, healthy individuals. Although adding to the combined effect of other SNPs, there was no significant independent effect of *COMT Val*158*Met* polymorphism on FA.

The studies showed a sex effect on *COMT* functioning. In particular, *COMT* activity in human prefrontal cortex is 17% higher in men than women (Chen et al., 2004). The sexual dimorphism in the normal brain could be due to interaction of estrogens with *COMT* activity. Estrogens inhibit *COMT* gene transcription (Xie, Ho, & Ramsden, 1999), while *COMT*, in turn, participates in the metabolism of estrogens (Worda et al., 2003). This sexual dimorphism is known to contribute to significant sex by genotype interaction effects on behavior and personality (Barnett et al., 2008; Chen et al., 2011; Gurvich & Rossell, 2015; Harrison & Tunbridge, 2008) as well as cortical thickness (Papaleo, Erickson, Liu, Chen, & Weinberger, 2012; Sannino et al., 2015).

To summarize, the studies reviewed above demonstrated negative associations between *Met* polymorphism and the white matter myelination. On the other hand, there have been reports of sexual dimorphism on *COMT* functionality. To the best of our knowledge, there have been no publications exploring possible relationship between *COMT* polymorphism and white matter structure in people of different sex. We hypothesized that *COMT* polymorphism would differentially impact on the strength of FA values in males and females. In particular, we expected to observe sexual dimorphism effects, that is, reduced integrity of white matter tracts in females with *Val* polymorphism (compared with their male counterparts) due to lower activity of *Val* polymorphism and putatively higher DA availability in females. In order to make our study comparable with the studies reviewed as above, we chose to group *COMT* genotypes according to the presence or absence of the *Met* allele (i.e., *Met*/*Met* + *Val*/*Met* vs. *Val*/*Val*).

We used a whole‐brain diffusion‐weighted imaging (DWI) tractography method with Tract‐Based Spatial Statistics (TBSS) analysis to examine influence of *COMT* polymorphism on the intrinsic brain networks in a sample of healthy adults. We chose FA as the measure of white matter structure, as it has been shown to have higher heritability than other DWI parameters, such as radial and axial diffusivity (Kochunov et al., 2010).

## 2 MATERIALS AND METHODS

### 2.1 Participants

The study included 82 right‐handed, White Caucasian, healthy volunteers (39 men; 43 women; mean age: 33.2 years; range: 19–54 years). The White Caucasian ancestry has been established by participants' self‐report. Exclusion criteria comprised the neuropsychiatric disorders in the participants or their first‐degree relatives, screened out by the Structured Clinical Interview for DSM‐IV (First, Spitzer, Gibbon, & Willimans, 1996). Participants were provided with full information about the study and they gave written consent according to declaration of Helsinki. The study was approved by Ethical Committee of King's College London, Institute of Psychiatry (London, UK).

### 2.2 Genotyping

We acknowledge the potential pitfalls associated with neuroimaging genetic studies looking at the effects of a candidate genes on brain function or structure (e.g., Bigos & Weinberger, 2010). These authors suggested a set of basic principles that would assure sufficient power and validity of such a study, for example, a rational approach to the selection of candidate genes, the careful control of nongenetic factors (i.e., sex, age, IQ), and selection of task paradigms that could be plausibly linked to the biology of the gene of interest. In our investigation of white matter connectivity, we chose a *COMT* gene that has been reported to be related to white matter integrity. We also modeled effect of sex as potential factor interacting with the effect of *COMT* polymorphism.

Cheek swab samples were collected and DNA extracted using the protocol developed at the Social, Genetic and Developmental Psychiatry Centre (King's College London, Institute of Psychiatry, London, UK) (Freeman et al., 2003). All participants were genotyped for *COMT* (*Val*158*Met*, rs4680). The genotype of the *COMT Val*158*Met* (rs4680) SNP was determined by allelic discrimination assay (C__25746809_50) based on fluorogenic 5′ nuclease activity: a TaqMan SNP genotyping assay was performed using an ABI Prism 7900HT and analyzed with Sequence Detection System software according to the manufacturer's instructions (Applied Biosystems, Warrington, UK). There was no statistical deviation from Hardy–Weinberg equilibrium for all the polymorphisms. The *COMT Val*/*Val* homozygosity was detected in 19 participants (12 males and 7 females) and 63 participants were *Met*‐carriers (27 males and 36 females).

### 2.3 DWI image acquisition

We acquired DWI that uses the water diffusion properties to investigate the microstructural architecture of white matter fibers (Basser, Mattiello, & LeBihan, 1994). FA is a parameter derived from DWI that provides a reliable surrogate of the white fiber microstructural integrity. All experiments were performed on a 3T scanner (Signa HDx, General Electric, Milwaukee, WI, USA). DWI was acquired on whole‐brain volumes with a single‐shot echo planar imaging sequence and an 8‐channel head coil. Imaging parameters were as follow: TE/TR = 104/17,910 ms, pixel size = 2.4 × 2.4, slice thickness = 2.4, 32 directions with *b* value = 1,300 mm^2^/s including four images with no diffusion weighting (*b* value = 0).

### 2.4 DWI data preprocessing

Processing of diffusion weighted images was performed using FSL FMRIB's Software Library (FSL, version 4.1.9, http://www.fmrib.ox.ac.uk/fsl) (Smith et al., 2004). Diffusion‐weighted data were corrected for eddy‐current distortions and simple head motion artifacts using affine registration to the first acquired volume. Then a binary brain mask image was computed for each subject using Brain Extraction Tool (BET) (Smith, 2002). Finally, FA maps were created from the diffusion tensor model employing the FDT diffusion tool.

### 2.5 Whole‐brain TBSS analysis

Voxelwise statistical analysis of the FA data was carried out using TBSS (Tract‐Based Spatial Statistics). TBSS is a useful tool for analyzing FA data as it improves sensitivity, objectivity, and interpretability through nonlinear registration with an alignment‐invariant tract representation (Smith et al., 2006). All subjects' FA data were aligned into a common space using the FNIRT (FMRIB's Non‐Linear Image Registration Tool) (Andersson, Jenkinson, & Smith, 2007), which uses a b‐spline representation of the registration warp field (Rueckert et al., 1999). Next, a mean FA image was created and thinned to create a mean FA skeleton which represents the centers of all tracts common to the group. Each subject's aligned FA data were then projected onto this skeleton and the resulting data fed into voxelwise cross‐subject statistics. A threshold of FA > 0.2 was applied to exclude areas of uncertain diffusion orientation and/or high intersubject variability.

A permutation program (FSL's Randomize) was applied to voxelwise statistics on skeletonized FA data for all subjects. A design and a contrast matrix were performed using General Linear Model (GLM Tools, part of FSL). Next, randomization procedure produced statistical maps so that each pixel value refers to the *p*‐value of the test. The threshold‐free cluster enhancement (TFCE) (Smith & Nichols, 2009) and 5,000 permutations options were applied.

The GLM analysis was performed where we have modeled main effects of the *COMT* (*Met*‐carriers vs. *Val* homozygotes) and sex, as well as interactions of *COMT* x sex. The age was considered as a covariate.

### 2.6 Volume‐of‐interest analysis

Although TBSS has been very popular approach for analyzing DWI data, the investigators also reported potential inaccuracies that are inherent in the FA skeleton projection and the substantial bias that it can introduce (see review, Bach et al., 2014). Therefore, in order to maximize the robustness and validity of our results we re‐evaluated the TBSS data by applying the volume‐of‐interest analysis (VOI).

According to Witelson's method, six parts of the corpus callosum were examined: rostrum, genu and rostral body, anterior midbody, posterior midbody, isthmus, and splenium (Witelson, 1989). By applying a nonlinear spatial normalization (computed during TBBS procedure), the VOI was co‐registered to the FA of each subject. Then, the mean FA was computed in the VOI for each subject. Finally, a statistical analysis was performed for each tract using Kruskal–Wallis test.

## 3 RESULTS

### 3.1 Whole‐brain TBSS analysis

**Figure 1:**
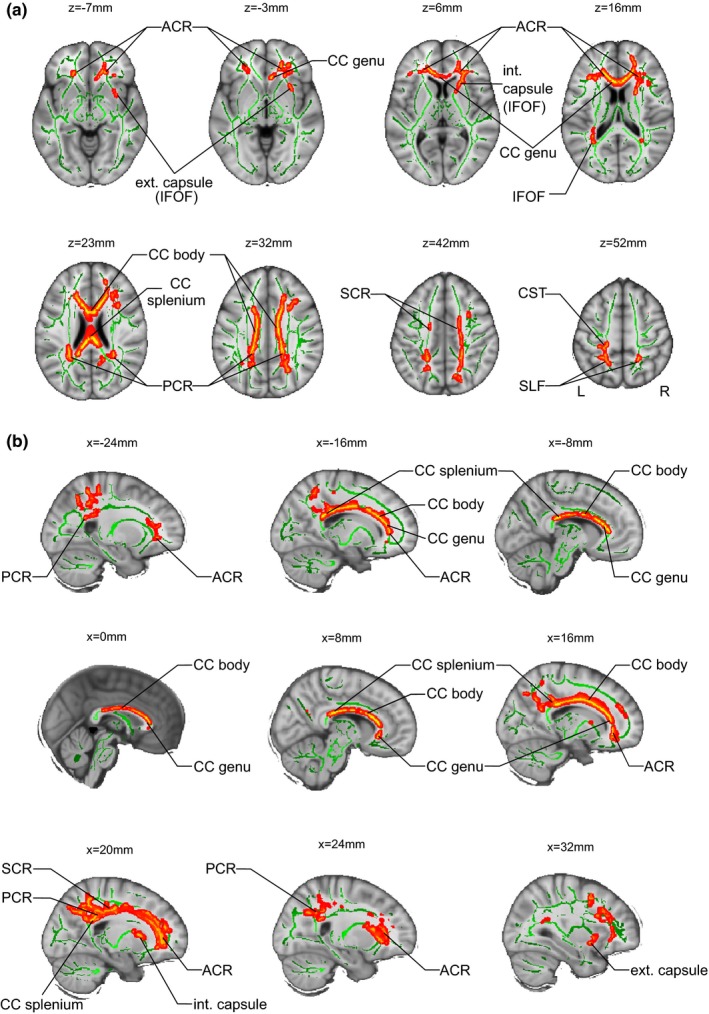
Genotype effect in male participants. FA maps. Fractional anisotropy (FA) skeleton is shown in green. To facilitate visualization, regions showing significant FA difference Males *Val* homozygotes > Males *Met* carries are highlighted in red‐yellow (*p* < .05 corrected for multiple comparisons), Regions showing significant FA difference are thickened using the tbssfill script implemented in FSL. Results are projected on the MNI 152 template and coordinates in MNI 152 space are indicated. L, left; R, right., Corpus Callosum; SLF, Superior Longitudinal Fasciculus; IFOF, inferior fronto‐occipital fasciculus; ACR, PCR, SCR, Anterior, Posterior, Superior Corona Radiata; CST, Cortico‐Spinal Tract. (a) Axial views. (b) Sagittal views

The GLM has not produced significant main effects of *COMT* polymorphism or sex on FA. However, a *COMT* effect on FA has been found to be moderated by sex in several regions. In particular, there were significant differences (all *p* < .05, corrected for multiple comparisons), with FA in male *Val*/*Val* > male *Met*‐carriers in the following major white matter tracts: the corpus callosum (CC) (genu, body and splenium) and the superior posterior portion of the left superior longitudinal fasciculus (SLF). The latter included horizontal fibers that connect superior parietal lobe (SLF‐I), angular gyrus (SLF‐II), and supramarginal gyrus (SLF‐III) with ipsilateral frontal and opercular areas. Significant differences were also observed in the internal capsule which may correspond to the inferior fronto‐occipital fasciculus (IFOF) and/or the uncinate fasciculus, in the superior part of the cortico‐spinal tract (CST), and the anterior, posterior, and superior parts of the corona radiata (ACR, PCR, and SCR, respectively). Results of TBSS are shown in Figure 1.

### 3.2 Volume‐of‐interest TBSS analysis

**Figure 2:**
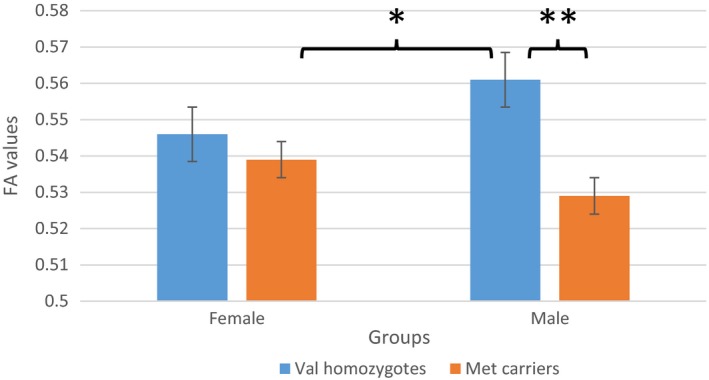
Genotype effect on fractional anisotropy in genu and rostral midbody in males and females (*p < .05; **p < .01). Blue bars: *Val* homozygotes; Orange bars: *Met*‐carriers

Using the VOI analysis to verify the results produced by the whole‐brain TBSS analysis, we found out that only CC data survived this test. Kruskal–Wallis H‐tests showed that, in males only, there was a statistically significant difference in FA values between the *Val* homozygotes and *Met*‐carriers in the following regions of CC: genu and rostral midbody, anterior midbody, posterior midbody, and isthmus. *Post hoc* tests (Mann–Whitney) demonstrated that in all these contrasts, male *Val* homozygotes had higher FA values compared with male *Met*‐carriers (Table 1, Figure 2).

**Table 1:** Fractional anisotropy (FA) in corpus callosum (means and standard deviations)

	Males (39)			Females (43)		
	*Val*/*Val* (12)	*Met*‐carriers (27)	*p*	*Val*/*Val* (7)	*Met*‐carriers (36)	*p*
Rostrum	0.487 ± 0.006	0.470 ± 0.004	ns	0.483 ± 0.007	0.480 ± 0.003	ns
Genu and rostral midbody	0.561 ± 0.006^a^	0.529 ± 0.006	<.01	0.546 ± 0.010	0.539 ± 0.004^a^	ns
Anterior midbody	0.595 ± 0.008	0.561 ± 0.007	<.01	0.586 ± 0.011	0.575 ± 0.004	ns
Posterior midbody	0.559 ± 0.009	0.518 ± 0.008	<.05	0.537 ± 0.015	0.541 ± 0.004	ns
Isthmus	0.624 ± 0.007	0.594 ± 0.007	<.05	0.611 ± 0.009	0.611 ± 0.003	ns
Splenium	0.669 ± 0.008	0.653 ± 0.006	ns	0.670 ± 0.005	0.666 ± 0.003	ns

a *Val*/*Val* males > *Met* females, *p* < .05.

## 4 DISCUSSION

Our study demonstrated sexual dimorphism of FA values in various regions of the CC that was associated with *COMT Val*158*Met* polymorphism. In particular, FA values were higher in *Val* homozygous males than in *Met*‐carriers males, whereas there was no genetic effect on FA values in females.

To the best of our knowledge, there have been only few studies directly investigating the relationships between *COMT Val*158*Met* polymorphism and white matter integrity.

Although Thomason et al. (2010) demonstrated higher FA values in genu of corpus callosum in healthy adolescents with *Val* homozygosity (compared with *Met*‐carriers), Kohannim et al. (2012) did not replicate these findings. The advantageous effect of *Val* homozygosity on white matter integrity has been reported by Liu et al. (2014), although in different brain regions.

Although we have not detected a main effect of genotype, we found that the *COMT Val*158*Met* polymorphism effect on FA in genu and rostral midbody of CC was moderated by sex, which adds an important dimension to the *COMT* functionality.

We have performed additional analysis to estimate potential effects of all variables of no‐interest on the hypothesized interaction—to account for all covariates (i.e., sex × age, comt × age, age × sex) as proposed by Keller (2014).

The additional analysis supported our original results, that is, FA in male *Val* homozygotes was higher than in male *Met*‐carriers in corpus callosum, SLF, IFOF, corona radiata, and CST at *p* < .05.

What are the implications of our finding of genotype‐ and sex‐related effects on CC fractional anisotropy? Corpus callosum contains WM fibers that connect the two hemispheres, and is involved in interhemispheric communication (Gazzaniga, 2000). Its known functions include interhemispheric exchange of sensory, motor, and cognitive information, and integration of inputs reaching one or both hemispheres (de Lacoste, Kirkpatrick, & Ross, 1985; Koch et al., 2011; Seltzer & Pandya, 1983; Wahl et al., 2007).

The literature of sex effect on CC size has been inconsistent. Several studies have failed to identify significant CC sex differences (e.g., Hasan et al., 2009; Fling et al., 2011; Westerhausen et al., 2011). A large sample study (Ardekani, Figarsky, & Sidtis, 2013) found that corpus callosum cross‐sectional area was larger in females after correcting for the confounding effect of brain size (which is usually bigger in males). Another large cohort study (Prendergast et al., 2015), which used the same segmentation of CC as our group (Witelson, 1989), demonstrated main effects for sex when predicting CC length, perimeter, and circularity (i.e., measure of whole CC shape). In particular, males' callosa were longer than females, with greater perimeter while controlling for intracranial volume. The authors reported that greater absolute corpus callosum genu values were observed in males until the fifth decade compared with females. Importantly, in a DTI study, Westerhausen et al. (2011) have found the sex effect on genu of CC with FA in males being higher than in females.

Our finding of a sex‐specific effect of *COMT* is in accordance with the literature on the role of *COMT* in sexual dimorphism. It has been known that estrogen reduces *COMT* enzyme activity, impacting on the effect of *COMT* genotype in women relative to men (Harrison & Tunbridge, 2008). Interestingly, sex‐dichotomous effects of functional *COMT* genetic variations on cognitive functions disappear after menopause (Papaleo et al., 2012).

There is dearth of data on sex‐specific effects of *COMT* genotype on brain white matter. An important study of Zinkstok et al. (2006) found that in young (18–35 years old) female but not male *Met* homozygotes, decreased white matter in left corpus callosum was associated with increasing age. The authors suggested that these differences could be related to estrogen‐related down‐regulation of *COMT* expression.

Our data showed that the difference between *Val* and *Met* activity may not be as pronounced in females compared with males—which should be taken into account when examining gene × sex interactions. For example, White et al. (2014) showed direct sex modulation of *COMT* effects on prefrontal activity. Male *Val* homozygotes exhibited significantly elevated activation compared with male heterozygotes. By contrast, corresponding *COMT* effects were less robust in females. This was interpreted as a compromised efficiency of prefrontal cortex in male *Val* carriers. However, these results could be explained in light of sexual dimorphism, for example, the Val effect in females could be inhibited (and appear comparable to *Met*) due to the impact of estrogen.

A differential effect of *COMT* polymorphism in males and in females might contribute to the inconsistencies in the literature on the association of *COMT* genotype with some behavioral phenotypes. In particular, *COMT Val* carriers demonstrated an impaired performance and higher number of errors in the Wisconsin Card Sorting Test compared to *Met*/*Met* individuals (Barnett et al., 2008; Joober et al., 2002; Rosa et al., 2004). Similarly, studies using working memory tasks in humans (or mice) showed that *COMT Val* carriers (and mice with artificially increased *COMT* activity) performed poorer compared to *COMT Met*‐carriers (and wild‐type mice) (Egan et al., 2001; Papaleo et al., 2012; Scheggia, Sannino, Scattoni, & Papaleo, 2012; Tunbridge, Harrison, & Weinberger, 2006). The other studies have reported no association between *COMT Val*158*Met* and performance in the WCST (Ho, Wassink, O'Leary, Sheffield, & Andreasen, 2005; Tsai et al., 2003) and the working memory task (Jacobs & D'Esposito, 2011). In light of our results of a sex‐specific effect of *COMT* on brain white matter (CC), it would be interesting to further examine whether these discrepancies potentially might be explained by sex effect on *COMT* functionality.

### 4.1 Limitations

Although our sample size was reasonably large, there were small numbers in some genotype groups, for example, Val homozygotes were represented by 12 males and 7 females. We have ensured that the Type 1 error has been controlled for by most rigorous two‐stage statistics, that is, the whole‐brain TBSS with subsequent nonparametric VOI analysis.

Another limitation of this study is that the individual plasma levels of estrogen were not assessed; therefore, we were unable to exclude any individual variation in estrogen levels. We assumed that in our group of healthy women (aged from 19 to 54), the levels of estrogen were within healthy range. Our conclusions on the inhibitory effect of estrogen on *COMT* activity have been based on existing literature.

## 5 CONCLUSIONS

To the best of our knowledge, this is the first study to examine the effects of sex and *COMT* polymorphisms on white matter connectivity in healthy adults. We found sex‐specific effect of *COMT* on brain white matter (CC): male *Val* homozygotes had significantly higher white matter integrity compared with *Met*‐carriers. This effect was absent in females where *Val* and *Met* groups had almost similar FA values in CC.

We believe that the importance of our results lies in highlighting the often overlooked joint effect of gene and sex on integrity of white matter tracts. Further research on larger samples is warranted that may shed more light on the sexually dimorphic effect of *COMT* on neuroanatomical structures.
